# A community-based intervention to improve screening, referral and follow-up of non-communicable diseases and anaemia amongst pregnant and postpartum women in rural India: study protocol for a cluster randomised trial

**DOI:** 10.1186/s13063-023-07510-x

**Published:** 2023-08-09

**Authors:** Jane Elizabeth Hirst, Nicole Votruba, Laurent Billot, Varun Arora, Eldho Rajan, Sudhir Raj Thout, David Peiris, Anushka Patel, Robyn Norton, Edward Mullins, Ankita Sharma, Stephen Kennedy, Vivekanand Jha, Devarsetty Praveen

**Affiliations:** 1 Nuffield Department of Women’s & Reproductive Health, University of Oxford, Oxford, UK; 2 The George Institute for Global Health, Imperial College London, London, UK; 3 The George Institute for Global Health, University of New South Wales, Kensington, Australia; 4 PGIMS Rohtak, Haryana, India; 5 The George Institute for Global Health, New Delhi, India; 6 The George Institute for Global Health, Newtown, Australia; 7 University of New South Wales, Kensington, Australia; 8 Prasanna School of Public Health, Manipal Academy of Higher Education, Manipal, India

**Keywords:** Maternal health, mHealth, Non-communicable diseases, Anaemia, Gestational diabetes, Preeclampsia, Community health workers, Rural India, Complex intervention, Clinical decision support, Hybrid type-2 implementation

## Abstract

**Background:**

Medical complications during pregnancy, including anaemia, gestational diabetes mellitus and hypertensive disorders of pregnancy place women are at higher risk of long-term complications. Scalable and low-cost strategies to integrate non-communicable disease screening into pregnancy care are needed. We aim to determine the effectiveness and implementation components of a community-based, digitally enabled approach, “SMARThealth Pregnancy,” to improve health during pregnancy and the first year after birth.

**Methods:**

A pragmatic, parallel-group, cluster randomised, type 2 hybrid effectiveness-implementation trial of a community-based, complex intervention in rural India to decrease anaemia (primary outcome, defined as haemoglobin < 12g/dL) and increase testing for haemoglobin, glucose and blood pressure (secondary outcomes) in the first year after birth. Primary Health Centres (PHCs) are the unit of randomisation. PHCs are eligible with (1) > 1 medical officer and > 2 community health workers; and (2) capability to administer intravenous iron sucrose. Thirty PHCs in Telangana and Haryana will be randomised 1:1 using a matched-pair design accounting for cluster size and distance from the regional centre. The intervention comprises (i) an education programme for community health workers and PHC doctors; (ii) the SMARThealth Pregnancy app for health workers to support community-based screening, referral and follow-up of high-risk cases; (iii) a dashboard for PHC doctors to monitor high-risk women in the community; (iv) supply chain monitoring for consumables and medications and (v) stakeholder engagement to co-develop implementation and sustainability pathways. The comparator is usual care with additional health worker education. Secondary outcomes include implementation outcomes assessed by the RE-AIM framework (reach, effectiveness, adoption, implementation, maintenance), clinical endpoints (anaemia, diabetes, hypertension), clinical service delivery indicators (quality of care score), mental health and lactation practice (PHQ9, GAD7, EuroQoL-5D, WHO IYCF questionnaire).

**Discussion:**

Engaging women with screening after a high-risk pregnancy is a challenge and has been highlighted as a missed opportunity for the prevention of non-communicable diseases. The SMARThealth Pregnancy trial is powered for the primary outcome and will address gaps in the evidence around how pregnancy can be used as an opportunity to improve women’s lifelong health. If successful, this approach could improve the health of women living in resource-limited settings around the world.

**Trial registration:**

ClinicalTrials.gov NCT05752955. Date of registration 3 March 2023.

**Supplementary Information:**

The online version contains supplementary material available at 10.1186/s13063-023-07510-x.

## Background and rationale {6a}

Over the past 20 years, significant progress has been made in India in reducing maternal and newborn deaths [1]; however, improvements have not been made evenly across the country and ensuring consistent quality care delivery across a vast population remains challenging. Globally, and in India, non-communicable diseases (NCDs) are women’s leading causes of death, and practical strategies are urgently needed to prevent premature mortality and morbidity [2, 3]. Complications that develop during pregnancy can identify women at increased risk for NCDs in the months and years following birth [4]. For example, hypertensive disorders of pregnancy (HDP), including gestational hypertension and preeclampsia, identify women at double the background risk for premature cardiovascular diseases [5], with around one in three women identified with an HDP having hypertension within the years immediately after birth, the leading risk factor for premature stroke [6]. Gestational diabetes mellitus (GDM), a form of glucose intolerance first detected in pregnancy and resolving with the birth of the baby and placenta, affects up to one in six pregnant people globally [7]. Those who develop GDM are at significantly increased risk of type 2 diabetes in the immediate postnatal years, with up to 50% having dysglycaemia within 5 years of the pregnancy [8]. Longer term, these women are also at increased risk of premature cardiovascular disease, a risk independent of type 2 diabetes [9].

### Pregnancy as an opportunity to improve women’s lifelong health

The potential for pregnancy care to be part of an integrated approach to NCD prevention has been recommended by the International Federation of Gynaecology and Obstetrics (FIGO) [10] and other leaders in women’s health [11–13]. Anaemia, HDP and GDM have been identified as priority areas to improve maternal and newborn outcomes in rural India [14]. Intensive diet and exercise programmes, lactation and medications can delay or prevent the onset of type 2 diabetes after GDM [15, 16]. Postnatal and annual screening for diabetes following a pregnancy affected by GDM is therefore recommended. Screening and management of hypertension and other cardiovascular risk factors, such as controlling weight, stopping smoking and measuring lipids, is also recommended after a pregnancy affected by HDP [17]. Little evidence exists for how this can be effectively translated into practice in low-resource settings.

Anaemia is another endemic problem in India, and eradication is a Government health priority [18]. During pregnancy, anaemia is associated with poor pregnancy outcomes, including maternal mortality, caesarean section, low birth weight, small for gestational age, preterm birth and perinatal and neonatal mortality [19]. Anaemia Mukt Bharat (AMB), launched in 2018, is the comprehensive national programme that aims to reduce anaemia across the country [18]. Despite investment and coordinated efforts, the programme targets for anaemia prevalence by 2022 are < 32% in pregnant women and < 40% in breastfeeding women are yet to be met.

### The SMARThealth programme

The George Institute for Global Health developed the Systematic Medical Appraisal, Referral and Treatment programme for cardiovascular disease prevention and management (SMARThealth) [20]. This global programme draws on innovative mobile-health technologies to promote evidence-based, clinical decision support systems. SMARThealth takes a health care ‘eco-system’ approach: (i) strengthening the capacity of primary health care workers and local communities; (ii) delivering fit-for-purpose electronic decision support systems and supportive technologies for self-management; and (iii) integrating with existing health system infrastructure. SMARThealth strengthens the skills of community health workers, known as ASHA (Accredited Social Health Activists) in India, to perform risk assessments for various conditions using an app deployed on a low-cost Android tablet. Data are stored securely on a cloud-based server with offline capabilities in areas of limited network connectivity. People at elevated risk of the screened conditions are electronically referred to the Primary Health Centre (PHC) for medical review. The PHC doctor has a complementary tablet app which communicates with the secure server and is provided with more complex decision support, including suggested medication management, based on national and international guidelines [21–25].

We have adapted the SMARThealth approach to improve the community-level identification, diagnosis, referral and management of women with anaemia, diabetes and hypertension during pregnancy and in the year after birth (SMARThealth Pregnancy) [26]. We demonstrated the acceptability and feasibility of SMARThealth Pregnancy in a pilot cluster randomised trial in four sites in rural India during 2019–2020 involving 200 pregnant women and 50 frontline health workers (unpublished work).

The SMARThealth Pregnancy programme evaluates both clinical and implementation effectiveness. A separate protocol will be published on the process evaluation, with detailed implementation outcomes and strategies.

## Objectives {7}

To determine if the SMARThealth Pregnancy complex intervention can improve women’s health in the year after pregnancy, specifically by decreasing the prevalence of anaemia by 9% and improving screening, referral and follow-up following a pregnancy affected by either anaemia, diabetes or hypertensive disorders of pregnancy. We also aim to evaluate our within-trial implementation strategy and identify the key determinants needed to support implementation beyond the trial phase.

Our hypotheses is that community-based screening, referral and follow-up in women with high-risk pregnancy conditions will improve health status at 12 months after birth and will be a low-cost and sustainable service delivery model.

## Trial design {8}

**Fig. 1:**
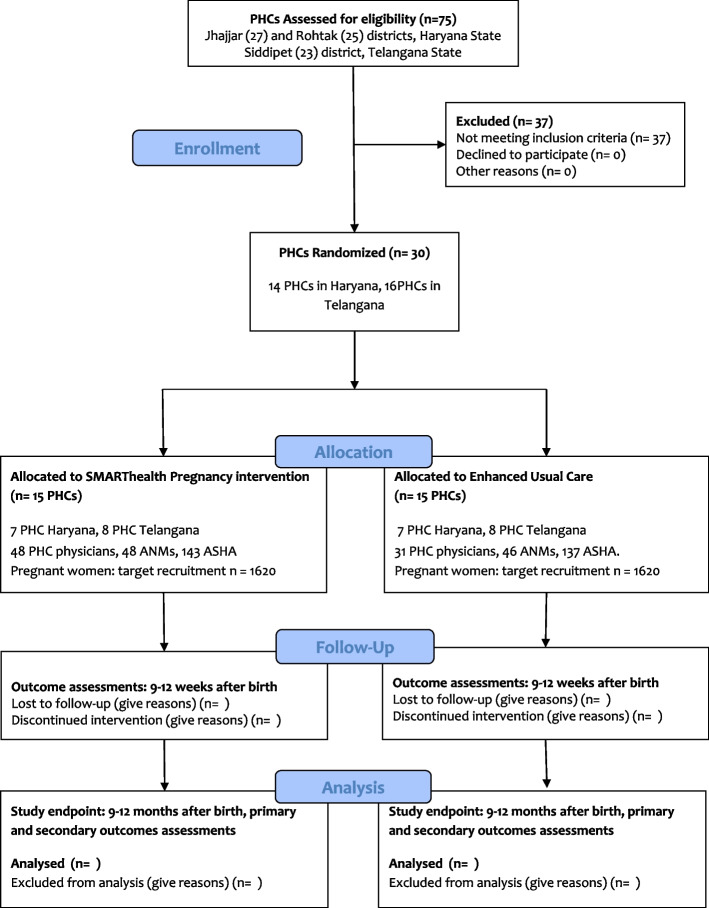
Consort diagram

A pragmatic, type 2 hybrid effectiveness/implementation, parallel-group cluster randomised superiority trial in two states in India (Haryana and Telangana). Thirty PHCs in Telangana and Haryana will be randomised 1:1 using a matched-pair design accounting for cluster size and distance from the regional centre. As the intervention is aimed at health workers working as teams (community health workers and PHC doctors), effectiveness is best evaluated through a cluster randomised trial with clustering at the PHC level (Fig. 1).

## Methods: participants, interventions and outcomes

### Study setting {9}

The study will be community-based and conducted in rural and semi-rural districts in India: Siddipet district, Telangana State, and Jhajjar and Rohtak districts, Haryana State. These have been chosen to represent two geographically, linguistically and culturally diverse populations.

Each PHC is responsible for primary care services for a population of around 30,000 people across several villages. We will select two villages per PHC as the study sites for the trial. All community health workers and PHC staff will be eligible to participate at each study site.

### Eligibility criteria {10}

#### Eligibility for primary health centres (PHCs)

PHCs will need to be located in one of the study districts, with the chief doctor (or equivalent) consenting to participate and support the programme for the duration of the study. In addition, at the time of enrolment, to be eligible, PHCs must have:

- ≥ 1 Medical officer
- ≥ 1 Laboratory technician
- ≥ 1 ANM
- ≥ 2 ASHAs
- The ability to administer iron sucrose infusions. Iron sucrose is the Government of India’s recommended treatment for moderate or severe anaemia in pregnancy that can be administered at the PHC level [18].

#### Exclusion criteria PHCs

PHCs with populations < 10,000 people, unwillingness to engage with the technology, or participation in a competing research study will be excluded.

#### Eligibility for women

Up to 5000 pregnant women will be screened until the sample size has been reached. Potentially eligible women will be identified by ANMs and ASHAs with the support of laboratory technicians. Women will be eligible if they are ≥ 18 years old. Women can be enrolled at any time during the pregnancy at or after 12 weeks of gestation, as determined by ultrasound scan, date of last menstrual period or best clinical estimate.

#### Exclusion criteria women

Women who decline participation; plan to move away and not return to the same village in the next 12 months; and do not speak or understand the local language (Telugu or Hindi). Women in India frequently return to their own mother’s village for the first few weeks after the birth. This will not be an exclusion criterion, provided they intend to return to the original village after this time.

### Who will take written informed consent? {26a}

We have based our approach to consent on a professional-cluster design approach proposed by Hemming and Eldridge [27, 28]. Consent will be collected at three levels:

1. *At the cluster level*, the study team will seek consent for each PHC from the designated clinical lead:
  a To be randomised
  b For all health workers affiliated with the PHC to participate in the study.
2. * From the health workers*, we will seek consent:
  a To participate in the study,
  b For data collection during and after the educational programme.
3. *From individual women*, a member of the study team will seek consent:
  a To participate in the additional study assessments at the start and end of the study.
  b For the study team to access and use their data for the purposes outlined in the informed consent form (supplementary file).

### Additional consent provisions for collection and use of participant data and biological specimens {26b}

This study does not involve the collection of biological specimens. As part of the consent form, participants will be asked if they agree for their data until that point to be included if they withdraw from the study. Participants will be asked permission for the research team to share relevant data with the international study team.

## Interventions

### The explanation for the choice of comparators {6b}

The study has been designed as a pragmatic trial to assess the impact of the intervention in a rural Indian context. In addition to current usual care, training will be held in all participating PHCs on screening for GDM using the oral glucose tolerance test, according to the Government of India recommendations [29] (enhanced usual care).

### Intervention description {11a}

**Fig. 2:**
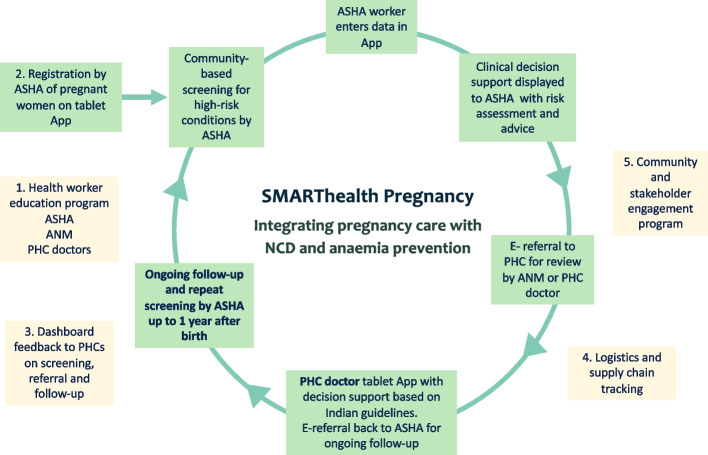
SHP intervention

SMARThealth Pregnancy is a complex intervention comprising five main components (Fig. 2):

1. A health worker education programme
  A 3-day training package for community healthcare workers (ASHAs) has been developed to improve knowledge of high-risk pregnancy conditions and raise awareness about the long-term implications of certain pregnancy-related conditions on women’s lifelong health. In addition, ASHAs are trained to perform point-of-care assessments, and how to use the SMARThealth Pregnancy app. PHC physicians will receive individual training on the SMARThealth Pregnancy programme, and on how to use the app.
2. The SMARThealth Pregnancy app
  The system was co-developed with end users with clinical decision support based on local guidelines.
  The app functions include:
  Secure storage of individual participant records
  Diary and schedule assistant to prioritise workload
  Facility to record blood pressure and haemoglobin results and track trends
  Facility to record iron folic acid compliance and supply
  Electronic decision support for blood pressure, haemoglobin, GDM screening, tetanus toxoid vaccination and deworming
  Electronic referral if indicated for PHC physician review
  Compliance monitoring for medication adherence (where relevant)
  Short health promotional videos aimed at women to be recorded in local languages about hypertension and diabetes, healthy diet and exercise, breastfeeding and future pregnancies
  The ASHAs will be reminded by the app to assess women periodically during their pregnancy and in the first year after birth, timed where possible with other health encounters such as scheduled antenatal visits and infant immunisations. ASHAs will be trained and equipped to perform point-of-care testing for common pregnancy complications. Anaemia will be screened using a point-of-care fingerstick device for haemoglobin measurement (TrueHb™). Blood pressure will be measured using an automated device validated for pregnancy (Omron™). ASHAs will ensure the woman is seated comfortably, with both feet flat on the floor and the cuff placed on the arm at the level of the heart. ANMs will be trained to administer the oral glucose tolerance test. The test is performed in the non-fasting state. ANMs will mix 75 g of glucose powder in one cup of water for the woman to drink. After 2 h, a fingerstick capillary glucose test will be performed. GDM will be diagnosed if the value is > 140 mg/dL.
  At the PHC, physicians will be issued a tablet computer with a version of the SMARThealth Pregnancy app designed to support guideline-based clinical decision-making in primary care setting. The main functions of the physician app are to generate recommendations for managing anaemia, hypertension and diabetes based on current Indian guidelines for pregnant and non-pregnant adults, as relevant to each woman.
3. The SMARThealth Pregnancy dashboard
  The SMARThealth Pregnancy study dashboard has been designed to provide real-time analytics and report generation that allows service oversight, including tracking of specified participant cohorts.
4. Supply chain and logistics support
  Through the SMARThealth Pregnancy app, we will monitor instances of stock-outs of iron folic acid tablets, iron sucrose, disposables for point-of-care testing, glucose for GDM screening and antihypertensive tablets. This information will be fed back to PHCs and district officials.
5. Stakeholder engagement
  Community stakeholder engagement events will be held throughout the study to increase awareness of the programme and the importance of high-risk pregnancy conditions, and co-develop pathways to implementation and sustainability after completion of the trial. We will also regularly update and engage with district officials and PHC physicians to ensure the programme addresses local priority needs and is seen as being owned by the communities.

Community stakeholder engagement events will be held throughout the study to increase awareness of the programme and the importance of high-risk pregnancy conditions, and co-develop pathways to implementation and sustainability after completion of the trial. We will also regularly update and engage with district officials and PHC physicians to ensure the programme addresses local priority needs and is seen as being owned by the communities.

### Criteria for discontinuing or modifying allocated interventions {11b}

There will be no predefined criteria for stopping the allocated intervention. If new national clinical guidelines are published during the trial, this may justify modification of the content of the intervention. The decision for modification will be made by the Steering Committee. The overall form of the intervention will remain unchanged.

Participants may withdraw from the trial at any time. Withdrawal will not negatively affect their ability to access usual care during pregnancy and after birth. As this study focuses on improving the woman’s health, stillbirth, neonatal or infant death will not exclude her from participating after delivery.

### Strategies to improve adherence to interventions {11c}

Study staff will support ASHAs by attending visits (with participant permission) and providing support for using the technology until the ASHA feels confident of performing assessments independently. Periodic quality control visits will be made to observe the intervention delivered by ASHAs and assessed by the study team. Every 6 months, the study team will conduct a 1-day refresher training session for the ASHAs.

Compliance with the intervention visits will be monitored centrally by tracking the visits recorded in the SMARThealth Pregnancy app. If trends of non-engagement across multiple participants from the same ASHA worker or PHC are noted, the study team will arrange a site visit.

For some women in India, it is traditional to move back to their mother’s village for the baby’s birth and stay there for up to 6 months. If the mother’s village is another intervention site, then visits will be transferred to the ASHA in the mother’s village, if it is part of the SMARThealth Pregnancy intervention. If the village is not an intervention site or is a EUC site, then visits will be replaced with phone calls, resuming face-to-face visits when the woman returns to her original village. Phone call follow-up was demonstrated to be acceptable and feasible for ASHA workers and women in the pilot study following COVID-19 disruptions.

### Relevant concomitant care permitted or prohibited during the trial {11d}

This is a pragmatic trial; hence, women will be free to attend any other health providers (including private health providers, traditional medicine practitioners and care providers from other government facilities) to seek additional care. These health utilisation patterns will be captured as part of the process evaluation.

### Provisions for post-trial care {30}

After the trial, any women with diabetes or hypertension will be referred to the local NCD services for ongoing management.

#### Theoretical underpinning for implementation

The Medical Research Council’s (MRC) framework for evaluating complex interventions [30] was used to inform the design of the overall study, intervention development, implementation and evaluation processes. Drawing on the findings of the pilot study and the experience of the collaborators, the study interventions were tailored and adapted for effective implementation and study delivery. The Reach Effectiveness Adoption Implementation Maintenance (RE-AIM) Framework, which has been widely applied to address challenges of combined effectiveness-implementation trials [31], has been used to guide the selection of implementation measures, alongside Proctor et al.’s [32] implementation outcomes typology.

The SHP2 trial applies a complex set of implementation strategies. The development of the implementation strategies was informed by the Expert Recommendations for Implementing Change (ERIC) project [33]. The ERIC compendium will also be used to identify implementation strategies that can help overcome arising issues that are affecting implementation during the trial. Due to the set-up of the trial, it was not feasible to randomise the implementation side of the trial (i.e. to allocate participants to different implementation strategies), and all participants in the intervention arm were allocated to the same strategies. We will examine the effectiveness of the implementation strategies used in the SHP2 trial in a separate process evaluation.

### Outcomes {12}

#### Primary outcome

The primary outcome will be the difference in the proportion of participants with anaemia, defined as haemoglobin measured using a point-of-care test < 12 g/dL at 12 months after delivery between the intervention and EUC clusters. Anaemia has been chosen as the primary outcome as it is the most common condition affecting women during pregnancy and breastfeeding across India. Measurement using a point-of-care device was chosen as this has been recommended in national guidelines and is a common method for diagnosing anaemia in rural settings, thereby reflecting real-world practice. The measurement will be performed by a member of the study team.

#### Secondary outcomes

Secondary outcomes will be collected across four areas:

1. Implementation outcomes
2. Clinical endpoints
3. Clinical service delivery indicators
4. Women’s wellbeing and mental health

(1) Implementation outcomes

Using the RE-AIM framework [31], we will report the following implementation outcomes (Table 1):

**Table 1:** Implementation outcomes to be assessed

RE-AIM dimension	Definition	Assessment measure	Data source	Timepoint
**Reach**	The number of people and percent of the target population who are impacted, and the extent to which those reached are representative and include those at most risk	Size of the target population	PHC records	Study end
		Number of women enrolled in the study and the number receiving the intervention	SHP app	Study end
		Comparison of characteristics of the individuals enrolled in the study to the target population	SHP app; district records	Study end
**Effectiveness**	A measure of the impact on health individual-level primary outcome including positive, negative and unintended consequences	The proportion of women with anaemia as measured by capillary test at 12 months after birth The proportion of women with follow-up blood pressure measurement or blood glucose tests during the 12 months after birth following a pregnancy affected hypertension or diabetes	Endline CRF	Study end
		The proportion of screening visits performed per protocol (maximum 7 SHP visits) The proportion of visits conducted in person and by phone The proportion of visits performed at the stated time in the protocol	Intervention group only: SHP app	Study end
		Self-report rating of health outcome (WHO-5 quality of life)	Endline CRF	Study end
		Self-report rating of health behaviour (WHO infant feeding questionnaire)	Endline CRF	Study end
		Quantitative comparison of outcomes across subgroups (family income, caste stratification, parity, rural/semi-rural, private/ government delivery) with qualitative exploration	Endline CRF, interviews and FGDs	Study end
**Adoption**	The number and percent of settings/providers/interventionists who participate, and the extent to which these are representative of those who the target population will use or visit	Number of PHCs invited and the number that participate at the start of the study	Baseline study information	Study initiation
		Number of participating PHCs at the end of the study	Endline study information	Study end
		Comparison of participating PHCs to non-participating PHCs in the district (size, location, number of pregnant women per year, sociodemographic indicators)	Local records	Study end
**Implementation**	Level of adherence to programme delivery as intended, including extent to which elements are implemented and/or adapted. Cost of delivering the programme	Ethnological observation to determine fidelity to SHP intervention delivery at different timepoints throughout the programme and different study visits	Interviews and FGDs	12 months and study end
		Proportion of intervention elements delivered as intended as measured by a checklist of essential elements	Checklists; Interviews and FGDs	12 months and study end
		Extent and number of adaptations to original protocol (fieldnotes, protocol amendments, app changes, additional trainings)	Checklists; Interviews and FGDs	Study end
		Cost of programme delivery (cost of intervention, training, materials, time-based activity costing)	Costing proforma	Study end
**Maintenance**	The degree to which the programme is sustained to the end of the trial (at the setting level) and to which the effects of the programme are maintained (at the individual level)	Level of intent to continue programme delivery (setting) as determined by in depth interviews with key stakeholders (PHC leads, other policy makers/local leaders) and Likert scale to community health workers and PHC doctors	Surveys; Interviews	12 months and study end
		Number of health workers trained to use the intervention Number of health workers who continue to use the intervention to study end Reasons for health worker drop outs	Intervention group only: SHP app, interviews	Study end

- *Reach*: The absolute number, proportion and representativeness of individuals that participated in the trial (both arms), and the intervention compared to the total population of pregnant women in the setting.
- *Effectiveness*: The impact of the intervention on clinical outcomes (described above); quality of life, measured using the EQ5D tool [34]; and economic outcomes (health expenses incurred during pregnancy, at the time of birth and in the postnatal first year, and incremental economic costs).
- *Adoption*: The absolute number, proportion and representativeness of health care workers who participated in the study compared to all health workers in the study locations. Qualitative work will explore reasons for non-participation or drop-out.
- *Implementation*: Fidelity to the intervention protocol for the trial and for the intervention itself and any adaptations during the trial. This will be measured assessing data completeness, and through qualitative methods with health workers.
- *Maintenance*: We will explore the system-level factors needed for institutionalisation of the intervention and level of intent to continue to programme in the study settings and more widely using key stakeholder interviews and focus group discussions, as well as policy discourse analysis around maternal health and digital health policy.

(2) Clinical endpoints
*Anaemia*: Haemoglobin will be compared between intervention and EUC clusters as a continuous variable (measured in grams per decilitre on point-of-care test) and as categories of anaemia using the WHO thresholds used for non-pregnant adults: no anaemia > 12.0 g/dL; mild anaemia 11.0–11.9 g/dL; moderate anaemia 8.0–10.9 g/dL; severe anaemia < 8 g/dL
*Diabetes*: Between the intervention and EUC, we will compare venous blood glucose following a 2-h, 75-g oral glucose challenge at 1 year after birth in participants diagnosed with GDM during pregnancy. Blood glucose will be assessed as a continuous measure (in milligrams per decilitre) and as categories of glucose impairment: Normal: < 140 mg/dl, impaired glucose tolerance (IGT): 140–199mg/dl, Diabetes: ≥ 200 mg/dl
*Hypertension*: Blood pressure (BP) will be compared between women who developed HDP. BP will be assessed as a continuous measure (systolic, diastolic and mean arterial pressure) in millimetres of mercury (mmHg) using a validated sphygmomanometer and as a binary category of hypertension (yes/no) defined as BP > 140/90 mmHg
(3) Clinical service delivery indicators
*Quality-of-care score*: We will measure differences between the intervention and EUC clusters using a quality-of-care score we have developed specifically for this study. The score comprises an equally weighted composite of clinical outcomes (anaemia, diabetes, hypertension) with measures of access and health professional visit frequency during pregnancy and in the first 12 months after delivery.
*Follow-up after anaemia, GDM or HDP condition*: We will compare how many women with anaemia undergo a haemogloblin test; how many women after HDP or GDM undergo BP measurement, and how many women after GDM undergo a glucose assessment (fasting or random capillary glucose, OGTT or HbA1c) within 6 weeks and 6 months and 12 months after birth.
(4) Women’s wellbeing, mental health, lactation practice
*Wellbeing*: The impact of the intervention on women’s general wellbeing, mental health. We will use locally validated mental health and wellbeing tools (EQ-5D-3L, PHQ9 and GAD7 [35]) to compare changes between baseline and at 12 months.
*Lactation*: is one of the few interventions for which evidence supports a positive impact on maternal and newborn long-term health following a high-risk pregnancy, specifically one affected by GDM [36]. We will assess whether the additional support provided through the intervention can improve lactation practice using WHO Infant and Young Child Feeding indicators [37].

### Participant timeline {13}

PHCs will be recruited and randomised before individual participant recruitment commences. After randomisation, ASHAs in the intervention clusters will attend the education programme to receive training on the clinical conditions, use of the point-of-care devices and SMARThealth Pregnancy app. Individual participants will be approached by their ASHA worker to participate after 12 weeks’ gestation. If they consent to participate in the study, a study team member will complete a baseline assessment, which includes questionnaires, blood pressure and haemoglobin measurement. A study team member will perform a mid-point assessment between 9 and 12 weeks after birth to capture events and healthcare utilisation including out-of-pocket costs around the time of birth. A study team member will conduct the final study visit 9–12 months after delivery, which will involve completing questionnaires, and measuring haemoglobin and blood pressure (Table 2).

**Table 2:**
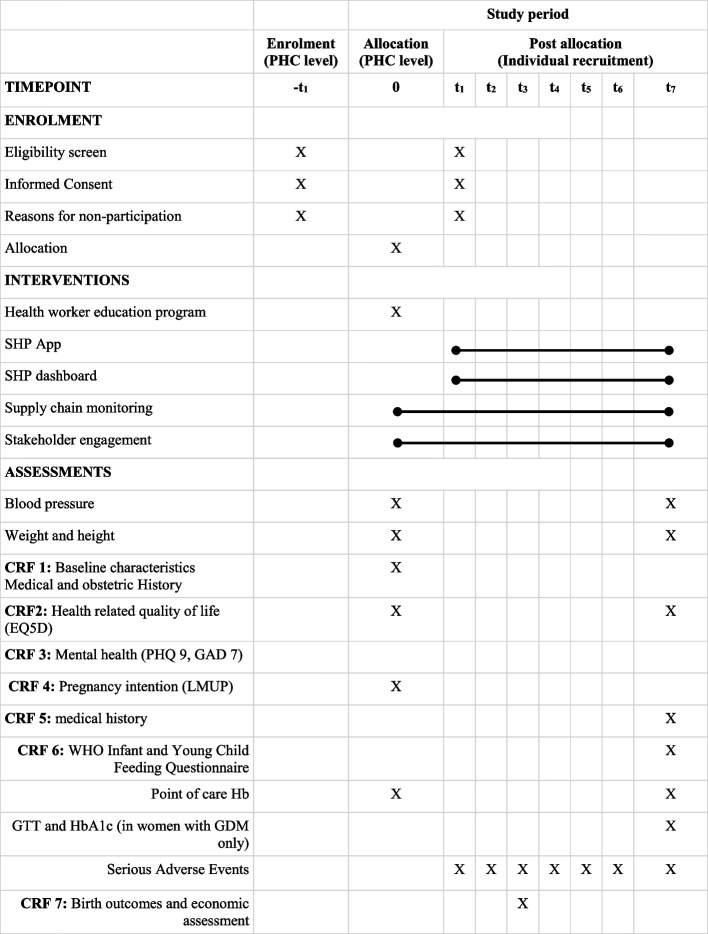
Participant timeline of assessments

PP = post partum. T_1_ = 12 weeks pregnancy; T_2_ = Third trimester; T_3_ = week one post birth; T_4_ = week 6 post birth; T_5_ = week 10 post birth; T_6_ = week 14 post birth; T_7_ = months 9-12 post birth. Visit intervals timed to be in line with Indian infant immunisation schedule

### Sample size {14}

The sample size was calculated using anaemia as the primary outcome. Three aspects were considered in determining the sample size:

1. Intracluster correlation
2. Cluster size and number
3. Variability in the primary outcome and estimated reduction that could be achieved by the intervention

These risks were balanced with operational considerations

#### Intracluster correlation

The nature of the intervention as a health system intervention precludes individual randomisation. Estimating intracluster correlation coefficients (ICCs) was based on the SMARThealth Pregnancy pilot study extrapolations [38]. Across the four sites in the pilot study, the ICC for anaemia was 0.019; thus, an ICC of 0.02 has been assumed for the study.

#### Cluster size and number

Clusters (i.e. PHCs) of various population sizes and birth numbers will be enrolled in the study. For operational reasons, a maximum cluster number of 30 is proposed, with two villages per cluster selected. Each village has one affiliated ASHA; thus, a minimum of 120 ASHAs will be needed.

#### Variability in the primary outcome and estimated effect size

The pilot study’s prevalence of anaemia during pregnancy was higher than the reported national levels, likely due to selection differences and differences in testing techniques (venous sample vs POC device used in the pilot study).

The prevalence of anaemia is slightly higher in non-pregnant than pregnant women (national estimate for India 53 and 50% respectively, NFHS 5 [39]). The national target for anaemia reduction is three percentage points per year as part of the Anaemia Mukt Bharat programme [18].

We estimate that our intervention will improve anaemia rates in these populations by 9% over 3 years (i.e. 53 to 44%). Determining a minimally clinically important threshold for anaemia is challenging. We selected the target decrease in anaemia as it was in keeping with the AMB programme [18], and to be an achievable and programmatically meaningful target with optimal support for anaemia prevention. Whilst anaemia is anticipated to decrease in the population because of the ongoing government programmes (including AMB), evidence from our pilot study is that compliance and implementation issues exist on the ground. Our intervention was designed to enhance delivery of the government programme and reaching of national anaemia reduction targets.

Given 30 clusters and an ICC of 0.02, 86 women per cluster are needed to detect a 9% absolute reduction (53 to 44%) with 80% power (a total sample size of 2580). Allowing for losses to follow-up of 20%, a sample size of 108 women per cluster is needed (a total sample size of 3240).

### Recruitment {15}

Each district will produce a list of all PHCs, with population size, number of ASHAs and number of births in the previous 12 months. After obtaining relevant permissions from the District Medical and Health Officer, the head of each PHC deemed eligible will be approached. Once they have consented to participate, the PHC will be randomised to either intervention or enhanced usual care.

Individual women will be identified by ASHAs affiliated with each PHC and by review of PHC records for women registered in the area for antenatal care. Women will be approached by the ASHA and given information about the study, then asked whether they agree to participate.

## Assignment of interventions: allocation

### Sequence generation {16a}

Before randomisation, each PHC will be paired with another similar PHC from the same state and with similar characteristics. The pairing will be based on:

- State (Telangana or Haryana)
- Distance in km to the district headquarters
- PHC total population size
- Local advice on population similarity (socio-economic, demographic, etc.).

Within each PHC pair, one PHC will be randomly allocated to intervention using a computer-generated randomisation programme. The other PHC will then be designated as the control. To ensure a 1:1 balance (the same number of PHCs allocated to intervention and to control) within each state, we will select an even number of PHCs in each state. Sixteen PHCs will be enrolled from Telangana and 14 from Haryana.

### Concealment mechanism {16b}

PHCs will be identified, and permission given to participate before allocation. After the central allocation of all clusters has been performed, each PHC will be notified of their allocation by the project manager. Given the pragmatic nature of this care-delivery trial, individual participants can only be recruited after allocation, and there will be no concealment before recruitment.

### Implementation {16c}

The allocation sequence will be generated by the study statistician (LB). Clusters will be enrolled before revealing the allocation. PHC physicians will be informed of their allocation by the Project Manager. Given the cluster design, participants will automatically be allocated to the group according to their PHC.

## Assignment of interventions: blinding

### Who will be blinded {17a}

Owing to the pragmatic nature of the trial, participants, care providers and outcome assessors will not be blinded to the allocation group. Data analysts, the Principal Investigator and the Steering Committee will remain blinded until the end of the study and database lock when the primary analysis will be performed.

### Procedure for unblinding if needed {17b}

All members of the clinical care team will remain unblinded throughout the study. As the statistician, Principal Investigator and Steering Committee are not directly involved in patient care in this study; there are no foreseeable reasons why the study would need to be unblinded.

## Data collection and management

### Plans for assessment and collection of outcomes {18a}

Independent, GCP-certified, trained, study team members will be involved in data collection at each stage. Data collection will occur on three occasions at both the intervention and control sites: baseline (after 12 weeks’ gestation), 9–12 weeks and then 9–12 months post-birth. All data will be captured on tablet computers using the SMARTHhealth app adapted for the clinical research forms (CRFs). Data will be de-identified, saved and stored on a secure server at the George Institute of Global Health, India. Strategies to ensure data quality include using published, validated questionnaires and the CRFs developed and user tested in the SMART Health Pregnancy pilot study. The field team supervisor will check approximately 10% of all CRFs for accuracy against source data or by direct interview with participants.

### Plans to promote participant retention and complete follow-up {18b}

Community education events will be held at intervention and control sites to promote the study and awareness of women’s health, anaemia, cardiovascular disease and diabetes. Refreshments and transportation costs will be provided to women at the baseline and annual study assessments.

ASHAs and PCPs will receive a small payment per participant to compensate for the time spent on the study. For women who move away from the study sites during their follow-up period, information on pregnancy complications and outcomes will be collected by phone where possible.

### Data management {19}

Data management will be performed in line with the study Data Management Plan. All baseline and outcome data will be entered digitally into the SMARThealth Pregnancy researcher platform. Access to data will be restricted to delegated study staff only and will require multifactor identification with a digital log kept of all logins. The trial manager and data managers will be primarily responsible for data quality. The trial manager will work with the project managers at both sites to ensure data quality, including completeness of data, data validation, data query management and training of database users. Monthly audits will be conducted to ensure the completion and quality of data captured.

### Confidentiality {27}

Throughout the study, all efforts will be made to maintain participant confidentiality. Each subject enrolled in the project will have a unique identifier (ID) of six digits. The first digits denote the study state (00 = Telangana, 01 = Haryana). The final four digits will denote the participant’s unique number, e.g. 00–0001 will be in Telangana and will be the first participant screened for both sites; 01–0002 will be in Haryana and will be the second participant screened for both sites. Participants who are screened but do not give consent or withdraw from the study will also receive a study ID to calculate intervention reach. Tablet computers used for data collection as part of the intervention and for the study assessments will be password protected with multifactor authentication. They will be used only by authorised users and stored in a secure location when not in use. Local data stored on mobile devices will be deleted daily following online database synchronisation. Participant-identifiable data will be collected in a separate database to the research data set and linked using a unique identifier (see above for details). This information will be held only for the study duration to enable follow-up by the study team.

### Plans for collection, laboratory evaluation and storage of biological specimens for genetic or molecular analysis in this trial/future use {33}

No biological samples will be collected or stored. Haemoglobin will be estimated using validated point-of-care devices. For the small number of women who develop GDM and require endline HbA1c, private local laboratories will process the samples with no sample storage.

## Statistical methods

### Statistical methods for primary and secondary outcomes {20a}

The primary analysis will be by intention to treat conducted at the participant level, using either random-effects models or generalised estimating equations adjusted for PHC clustering. We used a matched-pair design to balance cluster characteristics prior to randomisation, as recommended for community-based trials with a limited number of clusters [40]. Given the relatively small number of clusters, the analysis will not adjust for matching since this has been shown to provide unbiased estimates of the randomised intervention effect [41] and provide similar power to a matched analysis [40]. An unmatched analysis has the advantage that it can easily be conducted using participant-level data whilst allowing estimation of the intra-class correlation coefficient.

The final analysis is anticipated to occur 36 months after recruitment of the first participant. For the groups to be declared significantly different, the *P* value for the primary outcome must be ≤ 0.05. We will report the point estimate and confidence interval and present an assessment of the clinical relevance of our findings.

Baseline data will be presented for both groups. Continuous variables will be assessed for distribution, with appropriate statistical transformation (e.g. log or cubic splines) to correct for non-normality. Subgroup analyses will be performed by study location (Telangana or Haryana) to explore the effects of contextual factors on outcomes of interest. A detailed statistical analysis plan will be developed and publicly available before unblinding.

### Interim analyses {21b}

No interim analysis is planned as (i) part of the objective of the study is to determine if the intervention is sustainable over 12 months, and (ii) we are interested in the longer-term effects of the intervention on anaemia, blood pressure and blood glucose and early changes detectable between the groups may not be sustained.

### Methods for additional analyses (e.g. subgroup analyses) {20b}

Subgroup analyses will be performed for women with GDM and HDP separately and with both conditions. The analysis of secondary outcomes will be performed on all women reaching the study endpoint. A structured observation of health worker performance will be performed on a subsample of 200 women from 10 intervention and control PHC facilities to assess fidelity to the study intervention.

### Methods in analysis to handle protocol non-adherence and any statistical methods to handle missing data {20c}

The primary analysis population will include all participants with endline clinical assessment and haemoglobin measurement recorded. An intention to treat population will be defined as those participants who received at least one assessment during pregnancy and one after birth who also have a complete endline assessment. In case of substantial missing primary outcome data, sensitivity analyses based on multiple imputations will be performed.

### Plans to give access to the full protocol, participant-level data and statistical code {31c}

The full protocol, participant-level data and statistical code will be made available upon publication of the primary analysis as supplementary material. These will also be deposited in a data repository for future use.

## Oversight and monitoring

### Composition of the coordinating centre and trial steering committee {5d}

The coordinating centre will be in Hyderabad at the George Institute for Global Health. The Steering Committee comprises Indian and international experts in clinical trials, maternal health, health systems, implementation science and digital health. The trial Steering Committee will meet virtually or in person every 6 months to review trial progress, and be informed of severe adverse events, quality monitoring updates, modifications to the trial protocol, requests for data from other groups and plans for publications and dissemination.

The trial operations group will meet fortnightly (or more often if required) throughout the trial to ensure smooth day-to-day running and recruitment progress and provide organizational support to the field teams. The operations group comprises the project manager, the research staff directly involved and the Principal Investigators from the UK and India. The project manager will meet virtually or in person with each site manager weekly. The site managers have oversight responsibility for the field supervisors in their state.

### Composition of the data monitoring committee, its role and reporting structure {21a}

Given the pragmatic nature of this study and that there will not be an interim analysis, there will not be a data monitoring committee.

### Adverse event reporting and harms {22}

As the intervention aims to support the delivery of guideline-based antenatal and postnatal care already recommended in this setting, adverse events related to the intervention are not anticipated. That said, there may be unintended adverse effects from improved diagnosis of high-risk conditions (e.g. stigma), which could have physical or emotional consequences. All efforts will be taken by the study team to minimise potential harm. The project manager and field supervisors will identify and report any adverse events during the trial and promptly escalate these to the Principal Investigator (JEH). JEH will have the final say on whether such events are categorised as an adverse event (AE) or severe adverse event (SAE). She will be responsible for reporting any SAEs to the study sponsor and ensuring any actions are taken in the mandated timeframe. At the Steering Committee meetings, all adverse events will be reviewed. AEs and SAEs will be transparently reported in the primary publication, the final report to the REC and the final report to the study sponsor (The George Institute for Global Health, India).

### Frequency and plans for auditing trial conduct {23}

Trial conduct will be audited by the Data Management team at the George Institute at the start of recruitment and every 6 months, independently of the operational team. We will report annually to the study sponsor.

### Plans for communicating important protocol amendments to relevant parties (e.g. trial participants, ethical committees) {25}

Any protocol modifications will be reported to the George Institute India and Oxtrec ethics committees for minor or major amendment approval. Once approved, changes will be notified by the project manager to the site managers and field teams, who will inform the relevant health workers and participants. The trial register will be updated with any protocol amendments.

### Dissemination plans {31a}

The trial results will be communicated to the scientific and clinical communities through journal publications, and presentations at international and national conferences. Results will be communicated to local collaborators through written information and education sessions at the study end. Results will be shared with communities and women through local media and events.

## Discussion

This study will test the effectiveness and implementation of a community-based high-risk pregnancy screening and referral programme up to 1 year after birth. In rural India, as in many settings, women must attend antenatal appointments in a clinic or hospital to detect high-risk conditions. We hope to demonstrate that screening by community health workers in the woman’s village or home can be a practical approach to improving the detection of women with high-risk conditions, timely referral and evidence-based management. Uniquely, our intervention considers the impact of conditions detected during pregnancy on women’s longer-term health, with repeated assessments during the first year after birth. We hope to demonstrate that this is a practical approach to implement effective interventions for reducing the endemic problem of anaemia amongst women in India and engaging women who develop GDM or HDP with postnatal screening for diabetes and hypertension. In particular, the mixed quantitative and qualitative data collection in this hybrid effectiveness implementation study will help us analyse implementation outcomes and identify implementation strategies which are context-sensitive and can address implementation barriers of the programme.

The SMARThealth Pregnancy intervention aims to support government efforts to reduce anaemia rates through improved understanding by ASHAs and capacity to perform point-of-care haemoglobin testing. This is in keeping with AMB recommendations.

Our study has several strengths. The pragmatic nature of the trial will generate evidence of the effectiveness of strategies to improve the care of women at high risk of postnatal anaemia, type 2 diabetes or hypertension. Community follow-up after a pregnancy affected by these conditions is not routinely performed in rural India, which misses critical opportunities for prevention.

Our study will be conducted in two states with very different socio-cultural environments, which will improve the generalisability of our findings to other states in India. We are conducting this study as a hybrid effectiveness-implementation design. A separate protocol will be published on the planned implementation outcomes that we will capture through a detailed process evaluation. The analysis of implementation outcomes and strategies will inform the iterative development and steps needed for the eventual scale-up and adoption of the intervention more widely; alternatively, it will explain the factors that contributed to a null trial. As part of the process analysis, we will continue refining our logic model and assumptions regarding how the intervention works and the barriers to implementation, as well as the economic benefits and implications.

We acknowledge our study design has limitations. Owing to funding constraints, we can only involve community health workers from two villages affiliated with each PHC. Thus, the intervention will not be at the population level which might affect primary care physician engagement. We will explore this in detail in our process analysis. The nature of the trial design means there could be benefits above usual care in the control clusters. Participants in both study arms will have their haemoglobin tested and their blood pressure measured at the study visits. Ideally, after a high-risk pregnancy condition, follow-up would extend for life, which is clearly impractical and prohibitively expensive in the context of this study. We believe demonstrating engagement beyond 6 weeks after birth will be a strength. The nature of the complex intervention means that blinding of participants is not practical or feasible, increasing the likelihood of information bias. To minimise this, we will ensure that the trial statistician and investigator group remain blinded to allocation until the primary analysis has been performed.

Given the longitudinal nature of recruitment, not all potential participants will be pregnant before the study starts; therefore, randomisation must occur before participants are recruited. This could bias recruitment, as community health workers will know their allocation group when approaching pregnant women. However, in our pilot study, this was not the case: we achieved equal recruitment and retention rates across both groups.

In summary, we present a study protocol for a pragmatic, cluster randomised hybrid effectiveness-implementation trial to test the SMARThealth Pregnancy complex intervention in rural India. It is hoped that the trial will demonstrate the effectiveness of the intervention and implementation in reducing anaemia and improving, during and after pregnancy, the detection and follow-up of other high-risk pregnancy conditions.

## Trial status

The current protocol number is version 2.0, November 2022. The SMARThealth Pregnancy trial is currently recruiting. Recruitment commenced on 1 July 2022, and the trial is anticipated to run until 30 June 2024.

### Supplementary Information

**Additional file 1**. PARTICIPANT CONSENT FORM.

## Data Availability

The Principal Investigator, project manager, database manager and study statistician will have access to the final study dataset. The study team maintain the right to exclusive use of the data until the primary analysis is published or for 24 months after the end of the trial (whichever comes sooner). After this time, the data will be made publicly accessible as per the University of Oxford Open Access policy.

## Abbreviations

AE: Adverse event
AMB: Anemia Mukt Bharat: the Government of India programme for anaemia
ANM: Auxiliary nurse midwife
ASHA: Accredited Social Health Activist
CRF: Case record form
EQ5D: Euro-Quol Quality of Life measure - five domains
ERIC: Expert recommendations for implementing change
EUC: Enhanced usual care
FGD: Focus group discussion
FIGO: International Federation of Gynaecology and Obstetrics
GAD7: Generalised Anxiety and depression scale
GDM: Gestational diabetes mellitus
GCP: Good Clinical Practice for research
HbA1c: Glycated haemoglobin
HDP: Hypertensive disorder of pregnancy as per ISSHP 2018 guidelines
ICC: Intracluster correlation coefficient
ISSHP: International Society for the Study of Hypertension in Pregnancy
LMUP: London Measure of Unplanned Pregnancy
MRC: Medical Research Council, UK
NCD: Non-communicable diseases
NFHS: National Family Health Survey of India
OGTT: Oral glucose tolerance test
PHC: Primary Health Centre
PHQ9: Patient Health Questionnaire
SAE: Severe adverse event
SHP: SMARThealth Pregnancy
UKRI: United Kingdom Research and Innovation
