# Supplementary material for: A community-based intervention to improve screening, referral and follow-up of non-communicable diseases and anaemia amongst pregnant and postpartum women in rural India: study protocol for a cluster randomised trial
Source: Trials. 2023 Aug 9;24:510. doi: 10.1186/s13063-023-07510-x (PMC10410790; doi:10.1186/s13063-023-07510-x)
Supplement: Supplementary file 1 — Additional file 1. PARTICIPANT CONSENT FORM. [file 13063_2023_7510_MOESM1_ESM.pdf]

Informed consent materials

1. **ASHA workers**
2. **Women**
3. **PHC**

Version 1.0 30 July 2021

PARTICIPANT CONSENT FORM

SMARThealth Pregnancy 2

Ethics Reference:

Purpose of Study: The purpose of this study is to decrease anaemia and improve detection and follow-up of women with diabetes and hypertension during pregnancy and in the first 12 months after delivery.

		Please initial each box
1	I confirm that I have read and understand the information sheet version __________ dated ________________ for the above study. I have had the opportunity to consider the information, ask questions and have had these answered satisfactorily.	☐
2	I understand that my participation is voluntary and that I am free to withdraw at any time, without giving any reason, and without any adverse consequences or penalty.	☐
3	I understand that research data collected during the study may be looked at by designated individuals from the University of Oxford and The George Institute for Global Health where it is relevant to my taking part in this study. I give permission for these individuals to access my data.	☐
4	I understand that the information I give to the study may be used for other research studies in a way that I cannot be identified	☐
5	I understand that this project has been reviewed by, and received ethics clearance through, the University of Oxford Tropical Research Ethics Committee (OxTREC) and The George Institute Ethics Committee, India.	☐
6	I understand who will have access to personal data provided, how the data will be stored and what will happen to the data at the end of the project.	☐
7	I understand how this research will be written up and published.	☐
8	I understand how to raise a concern or make a complaint.	☐
9	I agree to be contacted by the study team by phone call, or through my ASHA worker, for follow-up during the study	☐
11	I agree to take part in the study	☐

Name of Participant	Date	Signature
Name of Witness	Date	Signature
Name of person taking consent	Date	Signature

PARTICIPANT CONSENT FORM
SMARThealth Pregnancy 2:
Ethics Reference:

Purpose of Study: The purpose of this study is to decrease anaemia prevalence and improve detection and follow-up of women with diabetes and hypertension detected in pregnancy and in the first 12 months after delivery.

S No		*Person taking consent to initial each box with permission of participant*
1	I confirm that I have read and understand the information sheet version __________ dated ________________ for the above study. I have had the opportunity to consider the information, ask questions and have had these answered satisfactorily.	☐
2	I understand that the participation of the Primary Health Centre (PHC) is voluntary and that the PHC is not obliged to enrol into the study.	☐
3	I understand that research data collected during the study may be looked at by designated individuals from the University of Oxford and The George Institute for Global Health where it is relevant to my taking part in this study. I give permission for these individuals to access data necessarily for this study from my health centre.	☐
4	I understand that this project has been reviewed by, and received ethics clearance through, the University of Oxford Tropical Research Ethics Committee (OxTREC) and The George Institute Ethics Committee, India.	☐
5	I understand who will have access to personal data provided, how the data will be stored and what will happen to the data at the end of the project.	☐
6	I understand how this research will be written up and published.	☐
7	I understand how to raise a concern or make a complaint.	☐
8	I agree for the PHC, its associated staff, and willing patient participants to take part in the study	☐

Name of Administrative head	Date	Signature
Name of witness	Date	Signature
Name of person taking consent	Date	Signature

PARTICIPANT CONSENT FORM
SMARThealth Pregnancy 2:

Ethics Reference:

Purpose of Study: The purpose of this study is to decrease anaemia prevalence and improve detection and follow-up of women with diabetes and hypertension detected in pregnancy in the first 12 months after delivery.

S No		*Please initial each box*
1	I confirm that I have read and understand the information sheet version __________ dated ________________ for the above study. I have had the opportunity to consider the information, ask questions and have had these answered satisfactorily.	☐
2	I understand that my participation is voluntary and that I am free to withdraw at any time, without giving any reason, and without any adverse consequences or penalty.	☐
3	I understand that research data collected during the study may be looked at by designated individuals from the University of Oxford and The George Institute for Global Health where it is relevant to my taking part in this study. I give permission for these individuals to access my data.	☐
4	I understand that the information I give to the study may be used for other research studies in a way that I cannot be identified.	☐
5	I understand that this project has been reviewed by, and received ethics clearance through, the University of Oxford Tropical Research Ethics Committee (OxTREC) and The George Institute Ethics Committee, India.	☐
6	I understand who will have access to personal data provided, how the data will be stored and what will happen to the data at the end of the project.	☐
7	I understand how this research will be written up and published.	☐
8	I understand how to raise a concern or make a complaint.	☐
9	I agree to be contacted by the study team by phone call for follow-up during the study	☐
10	I agree to take part in the study	☐

______________________	______________	______________________
Name of Participant	Date	Signature
Name of Witness	Date	Signature
Name of person taking consent	Date	Signature
